# Surgical Out-Patient Notes

**Published:** 1883-07

**Authors:** W. H. Harsant

**Affiliations:** Assistant Surgeon to the Bristol Royal Infirmary


### SURGICAL OUT-PATIENT NOTES.

BY

W. H. Harsant, F.R.C.S.,

*Assistant Surgeon to the Bristol Royal Infirmary.*

---

During the last four years and a half I have from time to time written down a few brief notes of cases in the out-patient department of the Bristol Royal Infirmary, and on looking them over I have selected the following as likely to be of some interest to the practical surgeon. They are simply a few cases noted here and there as the exigencies of out-patient practice would allow, and are by no means a complete record of the cases of each kind mentioned. One of the disadvantages in the treatment of hospital out-patients is their irregular and intermittent attendance: thus a case which requires regular treatment for twelve months or more, such as a case of congenital talipes or of constitutional syphilis, will attend for three or four weeks and will then be lost sight of, the consequence being that very little good has been done, and the surgeon has lost sight of a case in which he is interested. Some exceptions are fortunately met with, and among them several will be found recorded below where patients have regularly attended for a period of two years or more, thus enabling the effects of treatment to be steadily observed. Another disadvantage, common, however, to all hospital practice, is the difficulty of ascertaining a reliable family history; this can only be obtained by the patient's own account, and is frequently of the most unsatisfactory and unreliable character. In some of the cases of congenital syphilis mentioned below I have succeeded in getting a tolerably complete history, as far as it can be thus obtained, but in others nothing definite could be found out.

### FAMILIES WITH CONGENITAL SYPHILIS.

Although cases of congenital syphilis are so frequently met with in the out-patient room, and are for the most part so easily recognised, yet it is always interesting to inquire into their family history, and to ascertain how far the disease has involved other members of the same family. The following are some of the examples that have lately come under my care:—

*Case* 1 consisted of a family of children, several of whom have been brought to me at different times, and are still under my occasional observation. I first saw them in April, 1879. The mother was a healthy-looking woman, but had an ulcer on the calf of her leg which was probably syphilitic. She said her husband suffered from abscesses and swellings on his bones. She had had ten children, of whom only five were living, the others had died in early infancy, and it was interesting to note how the disease seemed to wear itself out with successive births. Thus her first child died at two months and her second at one month old. The third lived and was affected the most severely. The fourth and fifth children died young, as did also the seventh. The sixth was living and was under my care. The eighth was also brought to me, while the ninth and tenth were living and quite healthy. The three children who were affected have all been under my care, and all have well marked congenital syphilis. The eldest, aged 14, is stone deaf, and has been so for three years; he has keratitis in both eyes, and marks of old sores over the nasal bones; he has a fœtid discharge from the nose, his teeth are healthy, but there are fissures around the mouth. The next child, aged 10, has very depressed nasal bones, and a fœtid discharge from the nose; she has obstruction of both nasal ducts, for which I have slit the canaliculi and passed probes. Her teeth are healthy. The other child, aged eight, has keratitis, a flat nose bridge, and scars about the lips. They have all been treated with cod liver oil and tonics, and have been much benefited.

*Case* 2.—A baby, aged seven weeks, was brought to me on July 2nd, 1880. The mother had six miscarriages, then a living child, then another miscarriage, then this child. The other living child was said to have had a rash when three months old. This child was well until it was two weeks old when it had snuffles and a rash on the nates and genitals. When I saw it it was covered with an erythematous rash, especially on the genitals; the feet and hands were peeling; it had snuffles and constant diarrhœa, the motions being green. It was treated with mercurial ointment applied to the abdomen, and when I lost sight of it there was considerable improvement.

*Case* 3.—A girl, aged 10, came to me on February 3rd, 1879. The family history here was negative. She was the youngest but one of four children, the others being said to be healthy. The mother could give no history of syphilis in herself nor in her husband. She had had one miscarriage at six months, between the first and second children. When the patient was twelve months old she had a rose rash on the buttocks and genitals, two months before I saw her both eyes became affected. When she came to me she had a very old look, the upper central incisors were typically pegged and notched, the lower teeth were much broken, and there were several scars about the chin and angles of the mouth. Both eyes were affected with advanced keratitis, so that she could only just discern light. She was treated with syrup of the phosphate of iron, but only remained under treatment for three weeks, when I lost sight of her.

*Case* 4.—A baby, aged four months, was brought by his mother on Feb. 10th, 1879. He was the youngest of three children. The eldest had a rash on the buttocks when three months old, and also had snuffles; it has been delicate ever since. The second child also had a rash at about the same period, and has since been delicate. This child (the third) was the worst of the three. He was only two weeks old when he first had a rash on the buttocks, and snuffles; now when four months old he has a tubercular eruption on the buttocks, face and head. The nose is much depressed. There is constant diarrhœa, with green motions, and a general look of cachexia. I could get no history of syphilis in the father or mother. He was treated with mercurial ointment applied to the abdomen, and cod liver oil internally, and had improved much when I last saw him.

*Case* 5.—I. M., aged three months, was brought to me on Sept. 23rd, 1879. It is an eighth child, all the others being healthy except the seventh, which had a rash on the buttocks when three months old, but is now well. This child had a rash when a month old, which first appeared on the nates, but afterwards spread to the scalp, arms, &c. It is a puny, wasted baby, covered with a mixed rash, vesicular, pustular and roseolar. It was treated with mercurial ointment, applied to the abdomen on flannel, and rapidly improved. When I last saw it the rash had nearly disappeared.

*Case* 6.—M. A. S., aged four months, was brought to me on March 28th, 1879. The mother had been married 15 years, and had six children, of whom five were living; all were said to be healthy except the youngest. Her husband had contracted syphilis and had infected her two years before the birth of the last child. After this she had given birth to a still-born child, and then to the one which she brought to me. The baby had an erythematous rash on the buttocks, and well marked snuffles; it presented a very wasted appearance. The mother had a gumma on the forehead. The baby was treated with mercurial inunction, and the mother with iodide of potassium, and both were under treatment for a considerable time with much benefit.

*Case* 7.—In this case mother and baby are both under my care, and the syphilitic history is clear. The mother is a healthy-looking country woman, aged 33. Three or four months after marriage, when pregnant with her first baby, she had a severe attack of syphilis, with sores on the genitals, rash and sore throat. Her husband had syphilis before marriage, but thought he had quite recovered. The first child was still-born. The second died at two months, of wasting and diarrhœa. The next miscarried at six months. The fourth is now living, aged five years, and has been cut for stone in the Infirmary, but is otherwise healthy. The fifth child died at eight months, of wasting. The sixth is the baby under my care, aged three months. It was six weeks old when a rash appeared on the buttocks and genitals. It has snuffles, and presents a prematurely old look; there is an erythematous rash on various parts of the body, and occasional diarrhœa. It is being treated with gray powder internally, and mercurial inunction to the abdomen. The mother is taking iodide of potassium, and both are improving.

*Remarks.* It is a well-known fact that the syphilitic dyscrasia has a tendency to wear out and exhaust itself upon the first children. This *law of decrease*, as it has been termed, is well exemplified in cases 1, 2 and 7. It has even been said that the regular diminution of the influence of the poison expresses itself in successive children by the longer or shorter duration of intra-uterine life. Thus Diday mentions a case where the first child was born at 6 months, the second at 7, the third at 7½, the fourth at full time, but surviving only 18 hours, the fifth also at full time, and surviving six weeks, and the sixth, lastly, living four months before any treatment was adopted. Here it is almost an arithmetical progression. And even in those cases which at first sight appear exceptions to this law of decrease, an explanation may be sometimes found in the fact exemplified in case 6, where the parents contracted the disease subsequently to the birth of the healthy children, but previous to that of the diseased ones.

The first sign of congenital syphilis in infants is nearly always a rash on the nates and genitals. Is there any peculiar characteristic in this rash by which alone we can recognize it as syphilitic? Probably not, but in the large majority of cases a rose rash on the nates and genitals of a young baby is indicative of congenital syphilis. Intertrigo must of course be distinguished from it, also the simple rose rash which appears on the nates at the same time as on the rest of the trunk, but a rash met with on the genitals and nates exclusively in a child of six weeks or two months old is in my experience generally syphilitic, even where no mucous tubercles are to be found.

### LUPUS VULGARIS.

*Case* 1.—E. S., a healthy-looking girl of 15, came to me on Oct. 31st, 1882, with a patch of lupus on the right cheek. It was single, and measured 4 inches by 1 inch. It had existed for 5 years, and at different times various caustics had been applied. When I saw it the skin was ulcerated over the whole of it with irregular nodules representing the original tubercles scattered here and there. There was very little strumous history to be obtained; her father and mother were fairly healthy, and one sister has since been attending me for a sinus under the lower jaw, leading to necrosed bone. I scraped the lupus thoroughly with a Volkmann's spoon, under chloroform, and dressed the wound with boracic lint. By November 26th it had completely healed, leaving nothing but a healthy looking cicatrix. I ordered her cod liver oil and arsenic, which she took for some time. On December 23rd the upper part of the scar presented three or four freshly developed tubercles; they had not ulcerated, but presented a whitish summit, and were evidently spreading, so I scraped the upper part again as before, and by January 14th, 1883, it was quite healed. On March 7th she came to me again with several tubercles at the lower part of the scar; the upper part was then looking quite firm and healthy. I scraped the lower part thoroughly, and on April 3rd, when I next saw her, it was all quite well. On April 27th she came to me again, with three fresh tubercles scattered irregularly over the scar, so on May 3rd I scraped these, and when I last saw her the cicatrix was all quite firm and well.

*Case* 2.—B. W., aged 24, came to me first on April 28th, 1882. On the centre of her left cheek were two patches of lupus, the upper one measuring 3 inches by $\frac{1}{2}$ inch, and the lower one being about the size of a sixpenny piece. Both places consisted of an aggregation of tubercles without any ulceration of the skin. She informed me that she had twelve months before been under the care of Mr. Malcolm Morris, at St. Mary's Hospital, and that he had scraped the upper patch for her, and that she had applied some caustic application once a fortnight afterwards for several months. She had thought for a time that it was cured, but it was now rapidly growing again. I placed her under chloroform and scraped both places thoroughly, taking great care to destroy all the soft parts. It had quite healed in a fortnight, and I hoped it was cured, but she came to me again on February 28th, 1883, with the upper patch showing several fresh tubercles. I scraped it again, and it soon healed, and I have not seen her since.

*Case* 3.—M. M., aged 20, a dressmaker, was formerly in the Infirmary with severe ulcerating lupus affecting the nose and left cheek. She was admitted on April 7th, 1882, and presented a very hideous appearance, having lost nearly all the nasal cartilages from ulceration, and having also an ulcerating patch on the cheek. She was treated for three months with strong chromic acid applied to the parts every three or four days, together with various constitutional remedies, but without any benefit whatever. She came under my care on July 30th, 1882, and I at once scraped away the whole of the soft growth from the nose and cheek, causing thereby no doubt some further loss of substance, but much less than might be expected. She made a rapid recovery, and on August 20th, when I last saw her, it had quite healed, and she expressed herself as feeling better than she had done for years. Although I have not seen this patient since, yet I recently heard that she had some slight return of the affection in the nose; should she come under my care again I should unhesitatingly scrape it afresh.

*Remarks.*—There are three methods of treating lupus. 1st. By caustics or the actual cautery. 2nd. By linear scarification. 3rd. By erasion. I have had some experience of the first method, and I consider it most unsatisfactory, it is extremely tedious, painful, and altogether uncertain. The actual cautery is probably the best form, but the sore left by it is a longer time in healing and makes a worse scar than the erasion method, but the great drawback is that it destroys some of the healthy surrounding tissues as well as the growth.

The second method, by linear scarification, I have never tried; it is strongly recommended by Mr. Balmanno Squire, the advantage claimed being that no tissue whatever is taken away, but the whole remains to form the new cicatricial tissue. If it could be depended on to destroy the growth, it would, no doubt, be better than the erasion method, but I cannot think it would be so effectual as the removal of the growth.

The third, or erasion method, was introduced some years ago by Dr. Volkmann, of Halle, and has since been very widely practised, and has met with a chorus of praise.

Mr. Jonathan Hutchinson, in his paper on lupus, read before the Association at Cork in 1879, said, "The erasion treatment appears to give less pain, to be very efficient, and to leave a sore which heals rapidly and soundly. Its introduction will mark a new era in the treatment of lupus. It is so simple, so easy of employment, and has in it so little that is alarming either to surgeon or to patient, that it is likely in the future to be employed in the early stages whilst there is good hope of the complete cure of the disease."

It consists simply in scraping away all the succulent cell growth with a Volkmann's spoon. This little instrument will not destroy healthy skin, but when drawn over a patch of lupus it will remove all the diseased parts, leaving only the healthy surrounding tissue. The wound made by it heals very quickly, and the resulting scar is very soft and flexible. It is the most satisfactory mode of treatment we at present possess. I have practised it in about ten cases, and recurrence has taken place in only four or five. In most of the others, I presume, the cure has been permanent or I should have heard of them again.

### STRUMOUS SYNOVITIS OF THE ELBOW CURED BY REST AND SIMPLE TREATMENT.

*Case* I.—E. J., aged 15, a boot machinist, living at Kingswood Hill, first came to me on April 13th, 1881, and has been regularly under my care ever since. Her family history is good, parents being very healthy. She was always delicate and thin and has had occasional coughs. Her chest is very flat, but there are no physical signs of phthisis. She had suffered from synovitis of the right elbow for three months before I saw her. It commenced without injury, and was at first very painful and swollen. On examination I found the synovial membrane of the right elbow presenting the usual boggy semi-fluctuating feeling met with in pulpy disease. The movements of the elbow were almost absent, and any attempts at movement were accompanied with acute pain. The limb was at first fixed on an angular splint, and Belladonna ointment applied to the joint. Cod liver oil was given, together with iron tonics. The splint was kept on for three months, and then Scott's dressing applied. She came to see me every fortnight and I used gentle passive movements, directing her to keep it quiet in the intervals. She wore the Scott's dressing for six months, and since then various simple remedies have been used at different times, such as the pressure of a bandage, frequent shampooing, iodine paint, &c., whilst she herself has made constant passive movements. The result has been very gratifying, for the joint is now nearly well. She can extend the elbow almost to a straight line, while she can flex it sufficiently to "do her back hair," which is the most satisfactory test of the usefulness of a woman's elbow. The power of supination is imperfect, and this movement still gives her some pain, but the swelling has nearly all disappeared, and the usefulness of the limb is increasing every day. The muscles of the arm and forearm are somewhat smaller than on the other side, but are daily improving with shampooing.

*Case* 2.—E. C., aged 27, is a thin, weakly-looking young woman, a dressmaker, living in the city. She came to me on May 2nd, 1882, and is still under treatment. Synovitis of the left elbow commenced three months before I saw her, without any injury, and had continued ever since. There was scarcely any movement in the joint when I first saw her, but acute pain on attempts at movement. It was first fixed on an angular splint, and Belladonna ointment applied to the joint. On October 24th the splint was discontinued and Scott's dressing applied; even now there was scarcely any movement. This dressing was continued until December 19th, when it was replaced by an ordinary cotton bandage, which was taken off daily to admit of shampooing and passive movements. She has taken cod liver oil nearly the whole time, and has once or twice been to the country for a change of air. The improvement in the joint has been steady and marked. She can now (May, 1883) flex the joint so as easily to reach the mouth, but cannot yet do her back hair; she can extend it almost to a straight line; supination is very imperfect, but pronation is perfect. There is now no pain and very little swelling in the joint.

*Remarks.*—These cases seem to me very encouraging, as they show that pulpy disease of the elbow, in some cases at all events, has a tendency to recover if placed under favourable circumstances. Are we ever justified in excising the elbow joint for pulpy disease? I should be inclined to answer that we are, if time is of great importance to the patient but not otherwise. A case of synovial disease, which has existed in the elbow for three or four months, so as to prevent the movements of the joint, will almost certainly require two years rest for its recovery, and it may be considerably longer before the limb is fit for any hard work; but it is something to feel that one may hold out favourable hopes of recovery in these cases even after the lapse of so long a time. I shall be very reluctant in the future to advise excision of the elbow for pulpy disease unless the bones also are involved in the joint mischief.

### SUPPURATION IN THE ANTRUM CURED BY FREE DRAINAGE.

*Case* 1.—G. G., aged 16, came to me on January 16th, 1883. He was a healthy-looking boy, living at Melksham, where he had been under surgical treatment for some time. He had noticed a swelling of the right cheek for three years, which slowly increased in size. When I saw him he had a firm elastic swelling causing a prominence of the cheek, pressing downwards on the hard palate and projecting most prominently within the mouth just above the lateral incisor tooth. There was a small sinus here which occasionally discharged pus. The teeth were all apparently sound and none were absent, nor had he suffered from toothache. I could not ascertain any cause for the mischief. On March 8th, chloroform having been administered, I made a free opening into the swelling with a scalpel just above the alveolus within the mouth. The anterior wall of the antrum was so thin that the scalpel entered it readily, and I was able to make an opening sufficiently large to admit my little finger. I scraped the interior of the cavity with a Volkmann's spoon, and turned out a quantity of inspissated pus and granular *dèbris*. I then syringed the cavity with boracic lotion, and gave him instructions to syringe it himself daily. The swelling rapidly diminished in size, the opening gradually closed, and when I saw him on April 5th he was quite well.

*Case* 2.—W. B., a labourer, aged 35, came to me on May 14th, 1883, with a similar swelling of the right antrum which had existed three years. The upper lateral incisor tooth of the right side was broken off across the middle of the crown from an accident twelve years ago. He said he had frequently suffered much pain in this tooth. The swelling projected into the right nasal fossa as well as on to the cheek and hard palate, and there was a constant purulent discharge from the nose. Chloroform having been administered, I first extracted the damaged tooth, and then pushed up a trocar and canula through the hole thus made in the alveolus into the abscess cavity. I then inserted a drainage tube into the opening, and cutting the end off level with the adjacent teeth, allowed it to remain in. He came to see me daily, and I syringed out the cavity with boracic lotion through the drainage tube. The purulent discharge from the nose at once ceased and the swelling diminished. The presence of the drainage tube caused him no inconvenience, but I thought it best to remove it after three days. The sinus soon closed after this, and he is now quite well.

*Remarks.*—Abscess of the antrum is generally caused by disease of the root of one of the upper teeth extending through the thin plate of bone separating them from the cavity. The tooth involved is generally the first or second molar, but in the case recorded above it was the lateral incisor which caused the mischief. The crown of the tooth was fractured by an accident, opening the pulp cavity; disease extended to the fang, causing much pain; suppuration ensued, and the mischief extended to the antrum.

Abscesses in the antrum, if left alone, discharge themselves generally either through the nose or through the anterior wall of the alveolus; cases have even been recorded where they have discharged through the orbit; but in any case they seldom cure themselves in this way. It is nearly always necessary to make a free dependent opening, so as to allow them to drain freely. If a tooth be deficient, the opening may be made with advantage through the empty socket, but if the teeth are sound and perfect it is quite sufficient to make the opening above the teeth, as in my first case recorded above. Abscesses in the antrum are for the most part easy to diagnose, but it is not always easy to distinguish them from solid growths of the jaw. Thus a little girl, aged two years, was brought to me on January 20th, 1883, with a soft elastic swelling of the cheek, pressing downwards on to the palate and upwards to the eyeball, as well as forwards on to the cheek. It felt so soft and fluctuating that I was induced to puncture it, thinking it might be a cyst or an abscess. It turned out to be a cancerous growth, and the child was admitted into the house, and afterwards had the upper jaw excised by my colleague, Mr. Prichard.

### MISPLACED TESTICLES.

*Case* 1.—J. T., aged 12, came to me January 8th, 1883. Both his testicles were undescended and lying in the inguinal canals close to the external abdominal rings. Both were painful and very tender, and had been so for some months. He was unable to walk or even to stand without pain, but was most easy in the sitting posture. Sometimes he could not sleep at night owing to the pain in the testicles, but sometimes they were quite easy. There was no hernia on either side. The scrotum was very small and atrophied. I prescribed a preparation of Belladonna for local application, and advised rest; but I never saw him again.

*Case* 2.—This was a curious case of displaced testis, the result of an injury. S. G., aged 43, came to me on February 7th, 1883. He had fallen off a ladder the day before, and had alighted on to his perineum astride of some iron railings. One of the spikes had penetrated the posterior part of his scrotum, causing a large effusion of blood into the scrotum and perineum. When I examined him there was a large blood swelling in the perineum, and the left testis had escaped from the scrotum and was lying farther back embedded in the blood clot. The effused blood slowly disappeared, but the testis remained in the perineum. I last saw him on March 20th. All swelling had subsided and the punctured wound had healed. The left testis was still in the perineum, and could be moved backwards and forwards with the fingers. He suffered no inconvenience from it unless he sat astride anything, when he had to be careful to draw the testicle forwards before sitting down. But as he told me he was not likely to ride on horseback I advised him to leave it alone.

### CHANCRE ON THE LIP.

J. E., a healthy-looking young woman, came to me on January 23rd, 1879, complaining of a swelling on her upper lip. Patient was single and was a paper bag maker by trade. Five weeks ago she first noticed a pimple on the lip, then a scab appeared on the surface, and it gradually became hard and increased in size until it reached its present dimensions. A week ago a rash appeared on the neck and thighs. I questioned her somewhat closely as to any infection, and the only explanation she could give was that she had been "keeping company" with a young man and had frequently kissed him, but she did not know that he had any cracks or sores on his lip. When I first saw her the chancre was an inch long a little to the right of the centre of the upper lip. There was a scab over part of it, and well marked induration around the base. There was a large mass of indurated glands in the right sub-maxillary region, and a papulo-squamous eruption on the back of the neck and thighs. She was at once placed on mercurial treatment, beginning with inunction of ointment until the gums were touched, and afterwards on the red iodide of mercury internally; but the attack was a very virulent one, and only gradually yielded to treatment. She continued as an out-patient until September 2nd, taking mercury in some form nearly the whole time. The lip had recovered long before, but the rash and enlarged glands had not quite disappeared when I last saw her.

### CONGENITAL TALIPES.

About twenty cases of congenital talipes equino-varus have come under my care during the last few years, and I have treated all of them pretty much in the way recommended by Ogston in his paper in the *Edinburgh Medical Journal* for December, 1878. I look upon congenital talipes as being due to a form of arrest of development of the fœtus, in which the affected feet have never become properly unfolded, a view which was first brought prominently forward in this country by Hardie, of Manchester, in the *British and Foreign Medico-Chirurgical Review* for April, 1871, and which has since been so widely accepted. I cannot at all understand Stromeyer's view, that it is caused by a spasmodic contraction of the muscles on the side to which the foot is directed, nor Barwell's theory, that it is due to paralysis of the muscles on the opposite side, although, doubtless, both these causes prevail in *acquired* talipes. But in congenital talipes the causes which give rise to the faulty position come into operation at a period long anterior to that at which the nervous or muscular system could be sufficiently developed to produce it, and the condition of the muscles is a consequence and not a cause of the affection. Holding these views, I make it my endeavour first to mould the affected feet into as good a position as possible with the hands, either with or without chloroform, according to the age of the patient, and then to fix them in the improved position with plaster of paris bandages. The first point is to restore the *varus*, which may be always done as easily without the division of tendons as with. Then afterwards the *equinus* is attacked, and this may be doubtless facilitated by division of the tendo achillis. I believe it to be important to attack the disease as early as possible, for then the bones and cartilages are so much more readily moulded into proper position. I am aware that Ogston recommends waiting until the child is six or nine months old before commencing proceedings, on the ground of the tenderness of the skin in very young children, but in one of my cases I put the bandages on to both feet of a child a month old, and it has worn them constantly ever since without any bad effects, although it was the worst case I ever saw. It is always necessary to warn parents of the tedious nature of the treatment before commencing. Friends are frequently anxious that the offending feet should be "turned" by a cutting operation, believing that this is all the treatment that will be necessary, and in this opinion they are too often supported by their medical attendant. It cannot be too strongly insisted upon that the treatment of congenital talipes must be a gradual one. The distorted skin, muscles, ligaments, cartilages and bones, have to be gradually moulded to their proper shape and fixed there for a considerable time before they will retain their new position, and the treatment has to be kept up for some time after the child begins to walk or it will surely recur. The first time the plaster of paris bandage is applied I generally keep it on about six weeks, it is then removed and left off two days to allow the skin to recover, then another is put on and allowed to remain about two months, and then afterwards it is renewed every two months until the child is about two years old, when the foot will generally be found to have assumed its normal shape. The muscles will even then require constant shampooing for some time before they will reach their proper size and strength, as there is always considerable want of development of the muscles in these cases. A great deal of the success depends upon early treatment. I have never had much success when the child has reached the age of twelve months before the treatment has been commenced.

